# MRI-derived T stage predicts nodal positivity on PSMA PET/CT in high-risk primary prostate cancer

**DOI:** 10.3389/fnume.2026.1854064

**Published:** 2026-07-29

**Authors:** Michael Courtney, Robert Whiriskey, Ciaran Johnston, Niall Sheehy

**Affiliations:** Department of Nuclear Medicine, St. James’s Hospital, Dublin, Ireland

**Keywords:** MRI, nuclear medicine, prostate cancer, PSMA PET/CT, risk stratification

## Abstract

**Aim:**

We evaluated whether Magnetic Resonance Imaging (MRI)-Derived T Stage independently predicts nodal disease on Prostate-Specific Membrane Antigen (PSMA) PET/CT in patients with high-risk primary prostate cancer, and developed a predictive model for clinical decision support.

**Materials and methods:**

MRI-derived T Stage, Prostate-Specific Antigen (PSA), Gleason score, age and PSMA node status were analysed using univariable and multivariable logistic regression in 152 men with high-risk primary prostate cancer who underwent pre-treatment multiparametric MRI and PSMA PET/CT. Model performance was assessed with area under the receiver operating characteristic (ROC) curve (AUC), calibration plots, and decision curve analysis. Subgroup analyses evaluated Gleason 3 + 4 and 4 + 3 disease separately.

**Results:**

MRI T3b and T4 stages were independently and significantly associated with increased risk of PSMA-positive nodal disease (*p* < 0.001 and *p* = 0.016, respectively). T3a disease was not significantly independently associated with PSMA-positive nodal disease (*p* = 0.086). T3b remained an independent predictor even in Gleason 4 + 3 cases (*p* = 0.026; odds ratio ≈ 23.57). The final model (AUC = 0.863) was well-calibrated and demonstrated clinical net benefit on decision curve analysis. In our cohort, patients with PSA < 20 ng/mL, MRI-derived T2 disease, and Gleason score < 8 (including both 3 + 4 and 4 + 3 patterns) demonstrated a 0% rate of PSMA-positive nodal disease.

**Conclusion:**

MRI-derived T stage T3b disease is a strong independent predictor of PSMA-positive nodal disease. These findings support guideline-based staging criteria and support the integration of MRI-derived T Stage into PSMA PET/CT triage workflows.

## Introduction

Prostate cancer is the second most commonly diagnosed malignancy in adult men worldwide with 1.5 million new cases diagnosed in 2022 and significant burdens on global healthcare systems (1–3). Accurate staging of prostate cancer is essential for guiding treatment decisions, particularly in patients with high-risk disease who may benefit from more aggressive therapy or extended lymph node dissection (4). Local T-staging traditionally relied on clinical examination, including digital rectal examination, as recommended by current guidelines (5). However multiparametric magnetic resonance imaging (MRI) plays a central role in local staging, offering high spatial resolution for evaluating extracapsular extension and seminal vesicle invasion, and contributing to Tumour Node Metastasis stage (TNM) classification through assessment of local tumour (T) stage (6). However, MRI has recognised limitations in detecting nodal metastases due to its reliance on size and morphological criteria, which lack sensitivity for small or morphologically normal-appearing metastatic lymph nodes (7–9).

The advent of prostate-specific membrane antigen (PSMA) positron emission tomography/computed tomography (PET/CT), hereafter referred to as PSMA PET/CT, has revolutionised the staging of prostate cancer (10, 11). PSMA PET/CT has demonstrated superior sensitivity and specificity for nodal and distant metastases compared with conventional imaging modalities, including MRI and computed tomography (CT), even in patients with low prostate specific antigen (PSA) levels or small-volume disease (12, 13). PSMA PET/CT demonstrates high diagnostic accuracy and offers a non-invasive alternative that approaches the diagnostic reliability of histopathology (14, 15). Consequently, PSMA PET/CT is increasingly adopted in the initial staging workup of high-risk prostate cancer, particularly in jurisdictions where access is expanding (16–18).

Despite its growing use, access to PSMA PET/CT remains limited in many centres due to limited PET scanner availability, radiopharmaceutical availability and staffing constraints (19). Identifying pre-test clinical or imaging predictors of node positivity on PSMA PET/CT could help stratify patients and optimise imaging resource utilisation (20). Whether MRI-derived T stage (an established marker of local tumour extent) predicts the likelihood of nodal involvement on PSMA PET/CT remains underexplored (21, 22). While recent studies have suggested that more advanced T stage correlates with increased risk of extra-prostatic and nodal disease (23, 24), direct evidence linking MRI findings, specifically MRI-derived T stage, to PSMA-detected nodal disease remains sparse (25).

To address this knowledge gap, we conducted a retrospective cohort study of 152 patients with high-risk primary prostate cancer who underwent pre-treatment MRI and PSMA PET/CT. We investigated whether MRI-derived T stage independently predicts nodal disease detected on PSMA PET/CT and assessed the contribution of clinical variables such as PSA, Gleason score, and age. We also explored whether combinations of clinical and imaging features could define subgroups with low or high risk of PSMA-positive nodal disease. Our findings aim to inform clinical decision-making and potentially guide the selective use of PSMA PET/CT in the staging of high-risk prostate cancer.

## Materials and methods

### Study design and patient cohort

**Figure 1:**
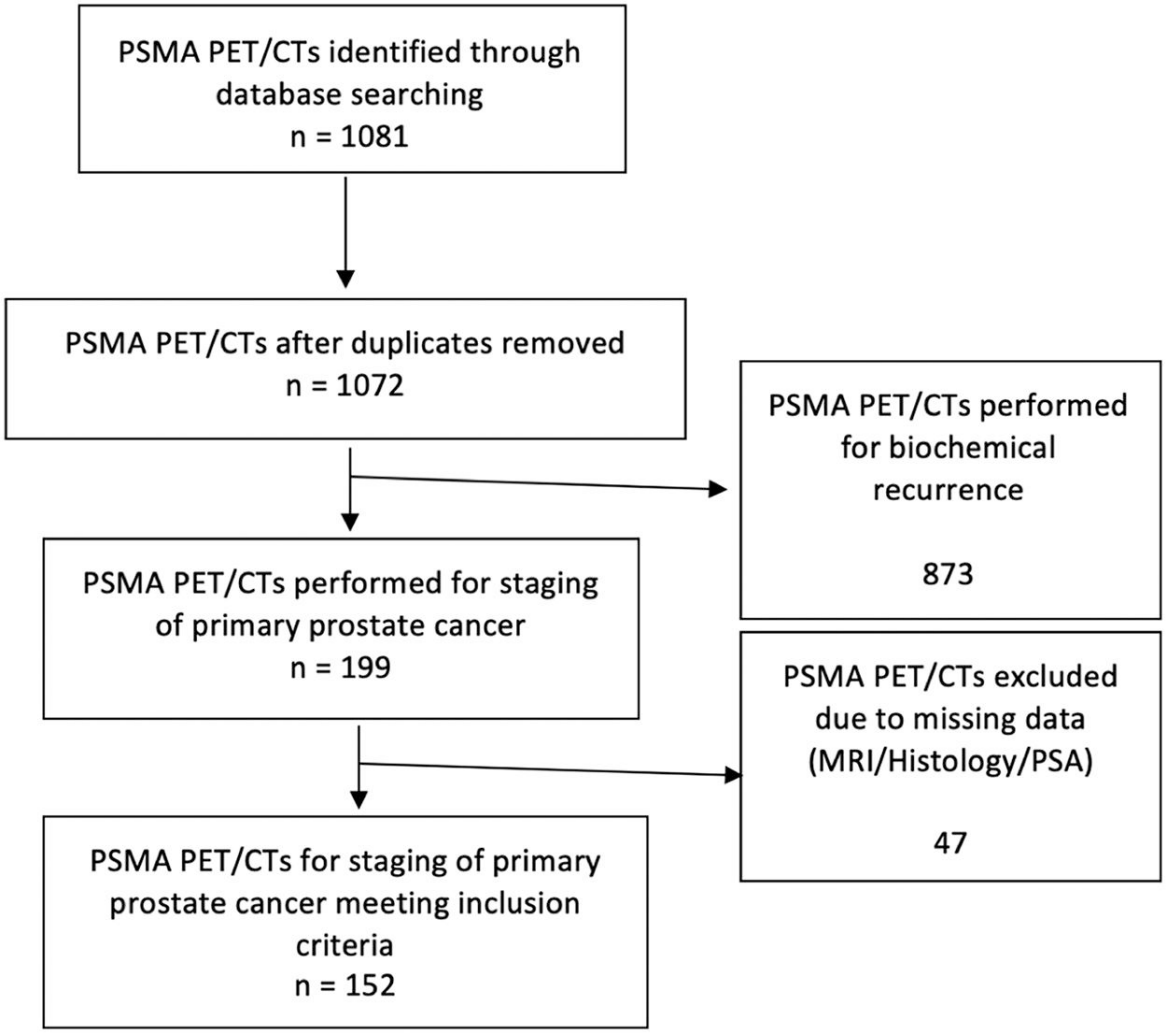
Cohort flow diagram.

This was a retrospective, single-centre cohort study conducted in accordance with institutional ethics approval. A total of 1,072 PSMA PET/CT studies were reviewed (Figure 1). Of these, 199 scans were performed for the primary staging of high-risk prostate cancer. The remaining 873 were excluded as they were performed for the investigation of suspected biochemical recurrence. Among the 199 staging scans, 47 cases were excluded due to absent data from the patients' electronic record (non-diagnostic MRI or MRI performed externally and unavailable for review or histology report not available). The final study cohort consisted of *n* = 152 patients. High-risk disease was defined based on established clinical criteria, including PSA ≥ 20 ng/mL, Gleason score ≥ 8, or clinical stage ≥ T3.

The study population was intentionally limited to patients referred with high risk primary prostate cancer undergoing PSMA PET/CT reflecting current guideline supported use of PSMA PET/CT for staging in this group. All imaging (both MRI and PSMA PET/CT) was performed before initiation of any oncological treatment. As such the cohort represents an appropriate high-risk pre-treatment population rather than an unselected staging cohort.

Demographic and clinical data, including age, PSA and Gleason score were extracted from electronic medical records. Histology and Gleason scores were obtained via pre-treatment core biopsy. During the study period, biopsies were performed using both transrectal and transperineal approaches. Imaging reports were reviewed by subspecialty-trained radiologists and nuclear medicine radiologists with more than 10 years of experience in reporting prostate MRI. MRI examinations were interpreted in accordance with Prostate Imaging Reporting and Data System (PI-RADS) v2.1 for lesion criteria and TNM 8th edition criteria for local staging (26) by subspecialty trained radiologists. All MRIs were performed on a 3 T scanner and all studies were reported using the updated 2019 PI-RADS guidelines. MRI studies were reviewed and tumours were categorised as T2 or less when confined to the prostate without radiological evidence of extracapsular extension or seminal vesicle invasion. T3a disease was defined by imaging features of extracapsular extension including capsular bulging or irregularity, capsular contact >10 mm, loss of the normal capsule-tumour interface, asymmetry or thickening of the neurovascular bundle, or frank extracapsular breakthrough. T3b disease was assigned by unequivocal contiguous invasion of the seminal vesicles characterised by low T2 signal extending from the tumour into the seminal vesicle lumen or wall with loss of the normal angle between the prostate base and seminal vesicle. T4 disease was assigned when there was radiological invasion of adjacent structures such as the bladder neck, rectum, levator ani, or pelvic sidewall. All cases were reviewed by two reporters, ambiguous/microscopic extracapsular extension and seminal vesicle invasion was only assigned when consensus agreement was reached with a third reporter in favour of definite extension, otherwise tumours were classified as T2. PSMA PET/CT nodal status was categorised as N0 (no nodal involvement), N1 (regional pelvic nodes), or M1a (non-regional distant nodal disease). PET/CT images were interpreted in consensus by experienced subspecialty trained nuclear medicine radiologists, with nodal involvement determined by visual PSMA avidity above background.

Patients were referred for PSMA PET/CT based on clinical T staging (digital rectal examination) in combination with PSA and biopsy Gleason score. As MRI was performed subsequently, some patients clinically staged as cT3 were reclassified as T2 or less on MRI-derived staging, contributing to the observed heterogeneity in MRI T stage within this high-risk cohort.

### Imaging protocols

All MRI scans were performed at our institution on a Philips Achieva 3 T MRI scanner (Philips Healthcare, Best, Netherlands) using a 32-channel phased-array pelvic coil with standardised multiparametric protocols using T2-weighted imaging in three planes, multi-b-value diffusion weighted imaging (b = 0, 500, 1,500 s/mm^2^). Representative T2-weighted sequence parameters include matrix size 320 × 320, slice thickness 3 mm, repetition time (TR) 3,000 ms, echo time (TE) 110 ms. Dynamic contrast enhanced imaging was not performed (27–29). PSMA PET/CT imaging was performed on a GE Discovery MI 4-ring digital PET/CT system with 64-slice CT capability (GE HealthCare, Chicago, IL, USA). The PET system uses lutetium-based scintillators and silicon-based LightBurst digital detector technology. Imaging was conducted using a standard institutional protocol following intravenous administration of [^68^Ga]Ga-PSMA-11, with a target administered activity of approximately 1.8–2.2 MBq/kg. Radiochemical purity was assessed for each production batch using high-performance liquid chromatography and was required to be >98% before clinical release. Image acquisition was performed approximately 60 min after radiopharmaceutical administration. Whole-body PET imaging was obtained from skull base to mid-thigh with patients positioned supine and arms raised where feasible. Low-dose CT was performed for attenuation correction and anatomical localisation, followed by three-dimensional PET emission imaging using time-of-flight reconstruction with an acquisition time of 3 min per bed position. PSMA PET reconstruction parameters utilised 2 iterations, 16 subsets, a 5 mm cut-off, standard filter, and 256 × 256 matrix.

### Outcome measures

The primary outcome was the presence of node positivity (N1 or M1a) on PSMA PET/CT. PSMA PET/CT was used as the reference standard for nodal status. Because nodal histopathology was not available for any patients, the model predicts PSMA-positive nodal disease rather than histologically confirmed nodal metastases. Secondary outcomes included stratification of PSMA-positive nodal disease by anatomical extent (N1 vs. M1a), and identification of clinical or imaging predictors of nodal involvement. Patients in the N1 and N1, M1a categories were labelled as “node positive” for the analysis of the primary outcome (presence of nodal disease), though a subgroup analysis of N1 vs. N1, M1a was also performed. Although the presence or absence of M1b and M1c metastases was recorded during data collection, no cases of M1b or M1c metastases were identified in the study cohort.

### Statistical analysis

All analyses were performed using R (version 4.5.0) within the RStudio environment. Continuous variables were summarised as means ± standard deviations or medians with interquartile ranges, as appropriate. Categorical variables were reported as frequencies and percentages. Baseline characteristics were compared between node-positive and node-negative groups using independent-sample t-tests or Wilcoxon rank-sum tests for continuous variables, and chi-square or Fisher's exact tests for categorical variables.

A multivariable logistic regression model was constructed to identify independent predictors of PSMA-positive nodal disease. Covariates included MRI T stage (categorised as T2 or less, T3a, T3b, or T4), Gleason score (7 vs. ≥8), PSA (continuous), and age (continuous). Covariates were selected based on established clinical relevance and recognised prognostic indicators of metastatic prostate cancer. Model performance was assessed using the area under the receiver operating characteristic curve (AUC), and calibration was evaluated using the Hosmer-Lemeshow goodness-of-fit test and bootstrapped calibration plots with 1,000 resamples.

Model performance was internally validated using bootstrap resampling (1,000 iterations); no external validation cohort was available, and all validation results therefore reflect internal validation only.

To assess the clinical utility of the model, decision curve analysis (DCA) was performed. In exploratory analyses, multinomial logistic regression was used to compare predictors of regional (N1) vs. distant (M1a) PSMA-positive nodal disease, using N0 as the reference group. A nomogram was generated from the final logistic model to facilitate individualised risk prediction.

All statistical tests were two-sided, and *p*-values <0.05 were considered statistically significant. All figures were generated in R (version 4.5.0) using RStudio and the following packages: ggplot2, rms, pROC, rmda, and forestmodel. The dataset was pre-processed using dplyr and tidyr.

This study was reported in accordance with the Transparent Reporting of a multivariable prediction model for Individual Prognosis Or Diagnosis (TRIPOD) statement for prediction model development studies with internal validation (TRIPOD Type 1b).

## Results

### Patient cohort

A total of 152 patients with high-risk primary prostate cancer underwent pre-treatment MRI and PSMA PET/CT. PSMA-positive nodal disease was identified in 51 patients (33.6%), including 26 (17.1%) with M1a nodal involvement. Baseline demographic, clinical, and imaging characteristics are summarised in Table 1.

**Table 1:** Baseline characteristics of the full cohort.

Variable	Category	Value
Total number of patients		152
Age (years)		67.2 (SD 7.0)
PSA (ng/mL)		13.0 (IQR 7.3–26.0)
MRI-derived T stage	T2 or less	33 (21.7%)
MRI-derived T stage	T3a	70 (46.1%)
MRI-derived T stage	T3b	41 (27.0%)
MRI-derived T stage	T4	8 (5.3%)
Gleason Score	7	53 (34.9%)
Gleason Score	8	32 (21.1%)
Gleason Score	9	64 (42.1%)
Gleason Score	10	3 (2.0%)
Gleason Score	Gleason ≥8	99 (65.1%)
PSMA PET/CT Nodal Stage	N0	101 (66.4%)
PSMA PET/CT Nodal Stage	N1	51 (33.6%)
PSMA PET/CT Nodal Stage	M1a	26 (17.1%)

PSA, prostate-specific antigen; MRI, magnetic resonance imaging; PSMA PET/CT, prostate-specific membrane antigen positron emission tomography/computed tomography; SD, standard deviation; IQR, interquartile range.

### Baseline characteristics by nodal Status

Patients with PSMA-positive nodal disease (*n* = 51) were younger, had higher PSA levels, more advanced MRI-derived T stage and were more likely to have Gleason ≥8 disease compared with node negative patients. MRI-derived T3b disease was markedly more prevalent among node-positive patients, while T2 or less was significantly more common among node-negative patients. Baseline characteristics, comparison and univariable analysis are summarised in Tables 1–3.

**Table 2:** Baseline characteristics by nodal status.

Variable	Level	Node negative (*n*)	Node positive (*n*)	*p*-value
Age		68.2 (SD 6.9)	65.4 (SD 7)	0.018
PSA		18.3 (SD 17.5)	38.4 (SD 82.1)	0.020
MRI-derived T stage	T2_or_less	31 (93.9%)	2 (6.1%)	<0.001
MRI-derived T stage	T3a	54 (77.1%)	16 (22.9%)	0.146
MRI-derived T stage	T3b	12 (29.3%)	29 (70.7%)	<0.001
MRI-derived T stage	T4	4 (50%)	4 (50%)	0.683
Gleason Score	7	43 (81.1%)	10 (18.9%)	0.010
Gleason Score	8_plus	58 (57.4%)	41 (80.4%)	0.029

Continuous variables were compared using Welch independent-sample t-tests or Wilcoxon rank-sum tests as appropriate. Categorical variables were compared using Fischer's exact test.

PSA, prostate specific antigen; SD, standard deviation; MRI, magnetic resonance imaging.

**Table 3:** Univariable logistic regression.

Variable	OR	CI	*p*-value
Age (per year)	0.94	0.89–0.99	0.019
PSA (per unit)	1.02	1–1.04	0.035
MRI-derived T3a vs T2 or less	4.55	1.35–20.9	0.025
MRI-derived T3b vs T2 or less	28.0	8.46–129	<0.001
MRI-derived T4 vs T2 or less	10.5	1.53–77.6	0.015
Gleason ≥8 vs 7	2.89	1.34–6.71	0.009

Univariable logistic regression analysis was performed using PSMA-positive nodal disease (N1/M1a) as the dependent variable. Odds ratios are presented for each variable individually without adjustment for covariates.

OR, odds ratio; CI, confidence interval; PSA, prostate-specific antigen; MRI, magnetic resonance imaging.

Reference category for MRI-derived T stage was T2 or less; reference category for Gleason score was Gleason 7.

Within the Gleason score 7 subgroup, PSMA-positive nodal disease was more frequent in patients with 4 + 3 disease compared with 3 + 4 disease, although this did not reach statistical significance. MRI-derived T3 or greater disease remained the dominant predictor of nodal positivity within this subgroup.

There were no cases of M1b or M1c disease identified in our cohort.

### Multivariable logistic regression

In multivariable analysis, MRI-derived T stage was the strongest independent predictor of PSMA-positive nodal disease, with T3b disease demonstrating the strongest association. Gleason ≥8 and younger age were also independently associated with nodal positivity, while PSA demonstrated a borderline association. Full multivariable results are shown in Table 4.

**Table 4:** Multivariable logistic regression predicting PSMA-positive nodal disease.

Variable	OR	95% Confidence interval	*P* value
T3a vs T2 or less	4.08	(0.98, 28.26)	0.086^†^
T3b vs T2 or less	36.45	(8.30, 267.55)	<0.001***
T4 vs T2 or less	14.09	(1.78, 151.01)	0.016*
Gleason 8–10 vs 7	3.04	(1.16, 8.69)	0.029*
PSA (per unit)	1.02	(1.00, 1.04)	0.083^†^
Age (per year)	0.92	(0.85, 0.98)	0.009**

Multivariable logistic regression was performed using binary logistic regression analysis with PSMA-positive nodal disease as the dependent variable.

Significance: ***p < 0.001.

** *p* < 0.01.

* *p* < 0.05.

† *p* < 0.1.

OR, odds ratio; PSA, prostate-specific antigen.

### Model performance and validation

**Figure 2:**
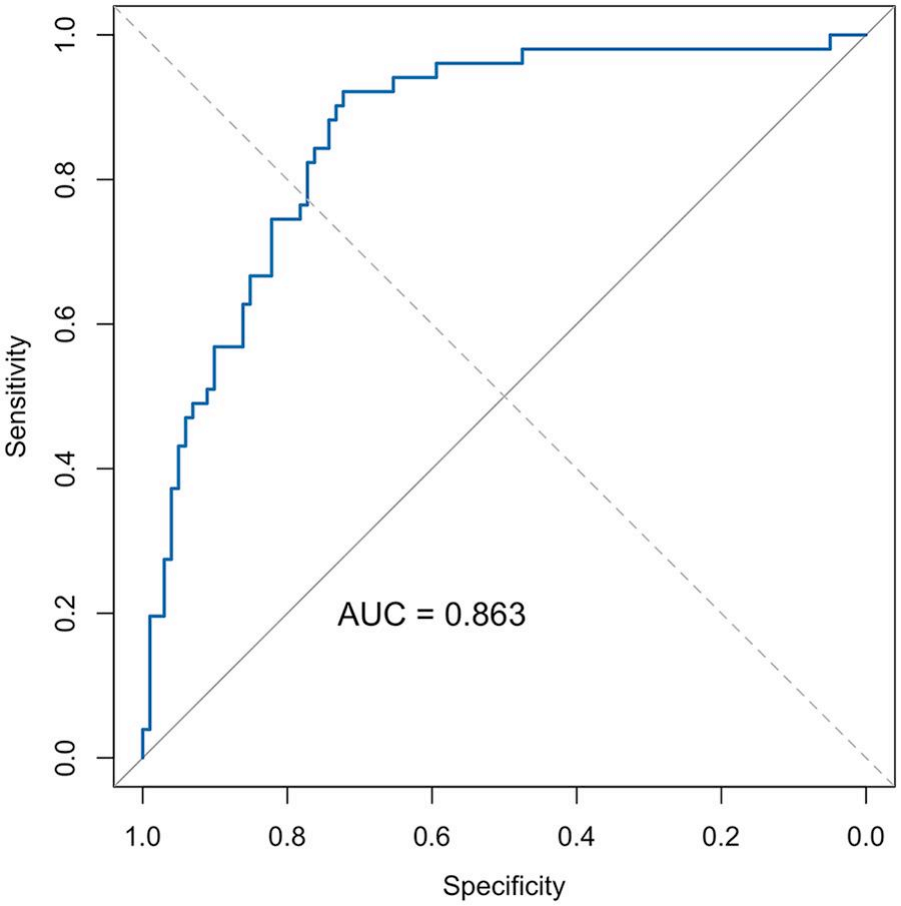
ROC curve: the ROC curve was plotted using the pROC package. Predicted probabilities from the final multivariable logistic regression model were plotted against observed PSMA-positive nodal status. The area under the curve (AUC) was computed with 95% confidence intervals (0.7947–0.916) via 2,000 bootstrap replicates.

**Figure 3:**
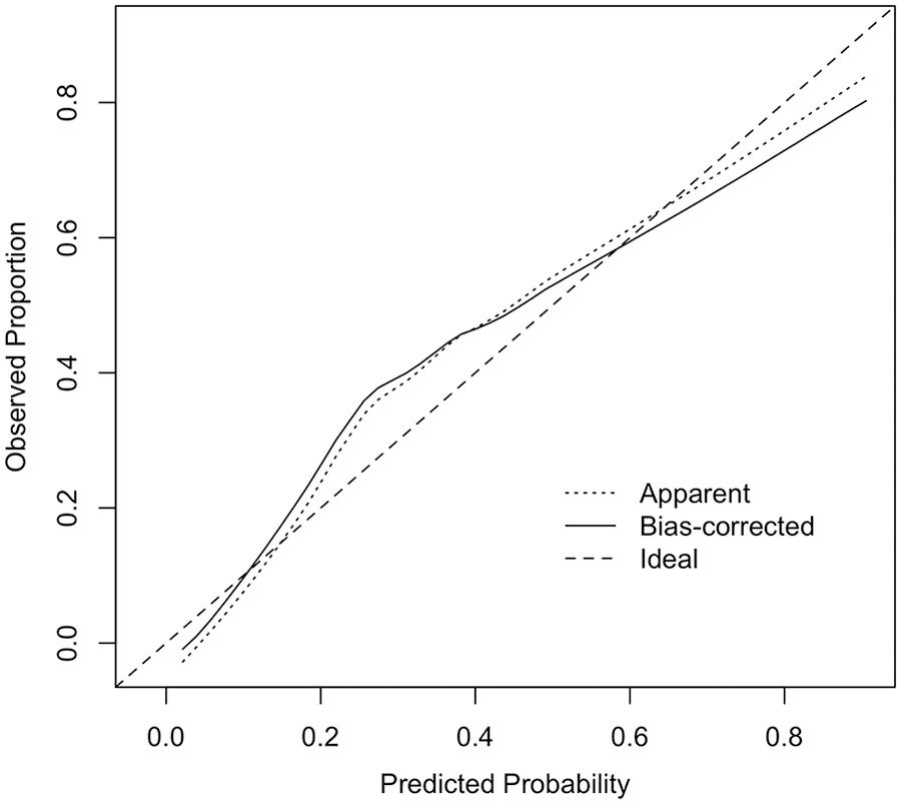
Calibration plot: Calibration was assessed using the calibrate function from the rms package with 1,000 bootstrap resamples. The plot visualises the agreement between predicted probabilities and observed outcomes, with a LOESS smoothed calibration curve and a reference diagonal.

The model demonstrated excellent discrimination, with an AUC of 0.863 [95% confidence interval (CI): 0.7947–0.916] (Figure 2). The Hosmer-Lemeshow test suggested good calibration (*p* = 0.78). Internal validation via 1,000-bootstrap samples showed mild model optimism, with an optimism-corrected Somers' Dxy rank correlation = 0.677 (corresponding to an optimism-corrected AUC of 0.839), R^2^ = 0.358, and calibration slope = 0.765, suggesting some degree of overfitting but overall indicating acceptable stability and calibration. The calibration plot confirmed close agreement between predicted and observed probabilities (Figure 3).

### Decision curve and calibration analysis

**Figure 4:**
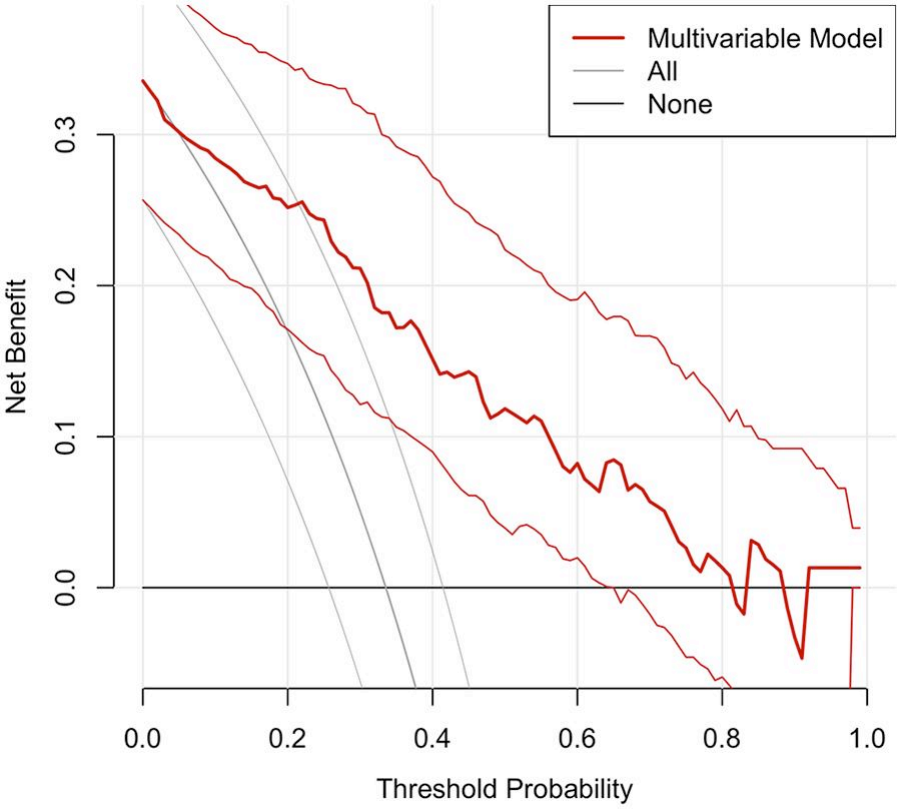
Decision curve analysis (DCA): Net benefit across a range of threshold probabilities was calculated using the rmda package. The DCA plot shows net benefit curves for the model, treat-all, and treat-none strategies to illustrate clinical utility.

Decision curve analysis demonstrated net clinical benefit for the multivariable model across threshold probabilities between 10% and 50%, supporting its potential utility in clinical decision-making (Figure 4).

### Subgroup predictive value analysis

To identify patients in whom PSMA PET/CT might be of limited utility, subgroup analyses of positive predictive value (PPV), negative predictive value (NPV), sensitivity, and specificity were performed. In patients with MRI-derived T stage of T2 or less, Gleason 7, and PSA <20 ng/mL (*n* = 10), no cases of PSMA-positive nodal disease were detected (PPV = 0.0%, NPV = 1.0), suggesting this combination represents a potential low-risk group in this cohort. Given the small sample size (*n* = 10), these findings should be considered exploratory and hypothesis generating. Other low risk combinations (e.g., T2 or less + PSA < 10 or Gleason 7 alone) showed lower specificity and less consistent performance. These findings are summarised in Table 5.

**Table 5:** Diagnostic performance of clinical subgroups for predicting PSMA-positive nodal disease.

Subgroup Definition	*N*	PSMA+	PPV	NPV	Sensitivity	Specificity
T2 or less	33	2	0.412	0.939	0.961	0.307
PSA < 10	57	12	0.411	0.789	0.765	0.446
PSA < 15	81	22	0.408	0.728	0.569	0.584
PSA < 20	97	28	0.418	0.711	0.451	0.683
Gleason 7	53	10	0.414	0.811	0.804	0.426
T2 or less & PSA < 10	14	1	0.362	0.929	0.980	0.129
T2 or less & PSA < 15	21	1	0.382	0.952	0.980	0.198
T2 or less & PSA < 20	25	1	0.394	0.960	0.980	0.238
T2 or less & Gleason 7	15	0	0.372	1.000	1.000	0.149
T2 or less & Gleason 7 & PSA < 10	6	0	0.349	1.000	1.000	0.059
T2 or less & Gleason 7 & PSA < 15	7	0	0.352	1.000	1.000	0.069
T2 or less & Gleason 7 & PSA < 20	10	0	0.359	1.000	1.000	0.099

Diagnostic performance metrics were calculated using PSMA-positive nodal disease as the reference standard. Sensitivity, specificity, positive predictive value (PPV), and negative predictive value (NPV) are reported for each clinical subgroup definition.

PSA, prostate-specific antigen; PPV, positive predictive value; NPV, negative predictive value.

### Multinomial regression for disease extent

In an exploratory multinomial logistic regression comparing patients with no PSMA-positive nodal disease (N0), regional PSMA-positive nodal disease (N1), and non-regional (distant) PSMA-positive nodal disease (M1a), we assessed whether clinical or imaging characteristics were associated with disease extent.

MRI-derived T stage of T3b was a strong independent predictor for both N1 disease [odds ratio (OR) 37.63, 95% CI, 4.22–335.94, *p* = 0.001] and M1a disease (OR, 34.42, 95% CI, 3.73–317.99, *p* = 0.002). T4 stage was significantly associated with M1a PSMA-positive nodal disease (OR, 29.44, 95% CI, 2.17–399.38, *p* = 0.011). The corresponding estimate for isolated N1 disease was unstable due to complete separation of the model arising from the small number of T4 cases and absence of isolated N1 events within this subgroup and should therefore be interpreted cautiously.

**Figure 5:**
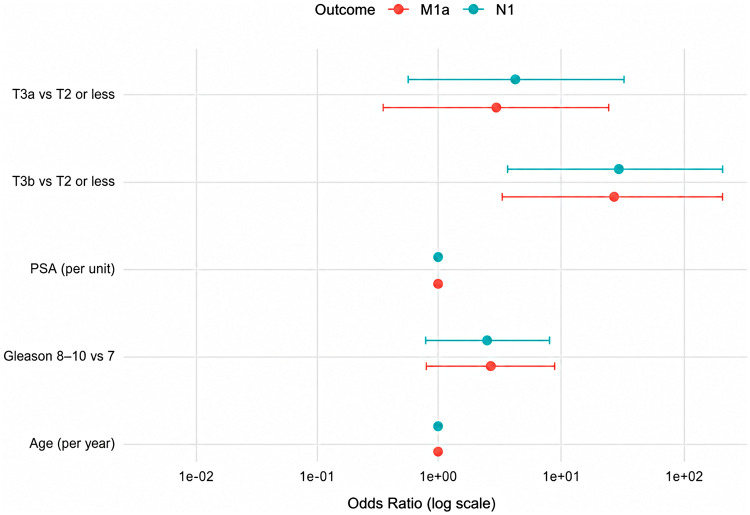
Forest plot of multivariable analysis: odds ratios with 95% confidence intervals for each covariate in the final logistic regression model were visualised using the forestmodel package. A log scale was used for effect sizes, and statistical significance was annotated.

Gleason ≥8 and increasing PSA demonstrated borderline associations with both N1 and M1a PSMA-positive nodal disease. Younger age was independently associated with increased risk of distant (M1a) PSMA-positive nodal disease (OR 0.90 per year, 95% CI 0.84–0.98, *p* = 0.012). The observed inverse association between age and PSMA-positive nodal disease should be interpreted cautiously. These findings are presented in Table 6 and Figure 5.

**Table 6:** Multinomial logistic regression (N1 and M1a vs N0).

Outcome	Variable	Odds ratio	95% CI	*p*-value
N1	T_stageT3a	4.82	(0.56, 41.59)	0.153
N1	T_stageT3b	37.63	(4.22, 335.94)	0.001**
N1	PSA	1.02	(0.99, 1.04)	0.147
N1	Gleason_8plus	2.79	(0.85, 9.19)	0.091^†^
N1	AGE	0.93	(0.86, 1)	0.061^†^
M1a	T_stageT3a	3.30	(0.36, 30.34)	0.292
M1a	T_stageT3b	34.42	(3.73, 317.99)	0.002**
M1a	T_stageT4	29.44	(2.17, 399.38)	0.011*
M1a	PSA	1.02	(1, 1.05)	0.057^†^
M1a	Gleason_8plus	3.36	(0.91, 12.42)	0.069^†^
M1a	AGE	0.90	(0.84, 0.98)	0.012*

Exploratory multinomial logistic regression analysis was performed using N0 disease as the reference category to evaluate predictors of regional PSMA-positive nodal disease (N1) and distant PSMA-positive nodal disease (M1a). Covariates included MRI-derived T stage, PSA, Gleason score and age. The T4 estimate for isolated N1 disease demonstrated complete separation due to sparse subgroup events and was therefore inestimable and has been omitted from the table.

Significance:

** *p* < 0.01.

* *p* < 0.05.

† *p* < 0.1.

CI, confidence interval; PSA, prostate-specific antigen.

### Nomogram-based risk estimation

**Figure 6:**
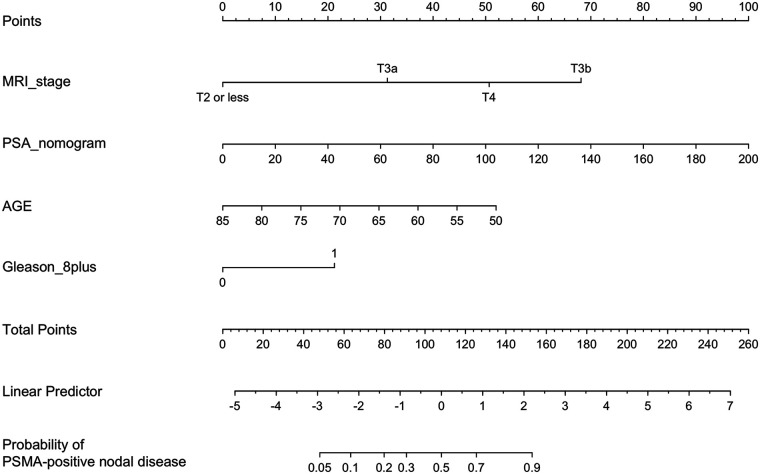
Nomogram: a nomogram was constructed using the nomogram function from the rms package based on the final multivariable logistic model. Point values for each predictor were assigned proportionally to their relative contribution to PSMA-positive nodal disease risk, and total points were mapped to predicted probability.

To facilitate individualised clinical risk assessment, we developed a nomogram incorporating MRI-derived T stage, Gleason score, PSA, and age to estimate the probability of PSMA-positive nodal disease (Figure 6). Points are assigned to each variable based on their relative contribution in the multivariable model, allowing user-friendly estimation of PSMA-positive nodal risk. We calculated the ten clinical profiles in the cohort with the lowest predicted risk of PSMA-positive nodal disease, all under 2.5%.

**Figure 7:**
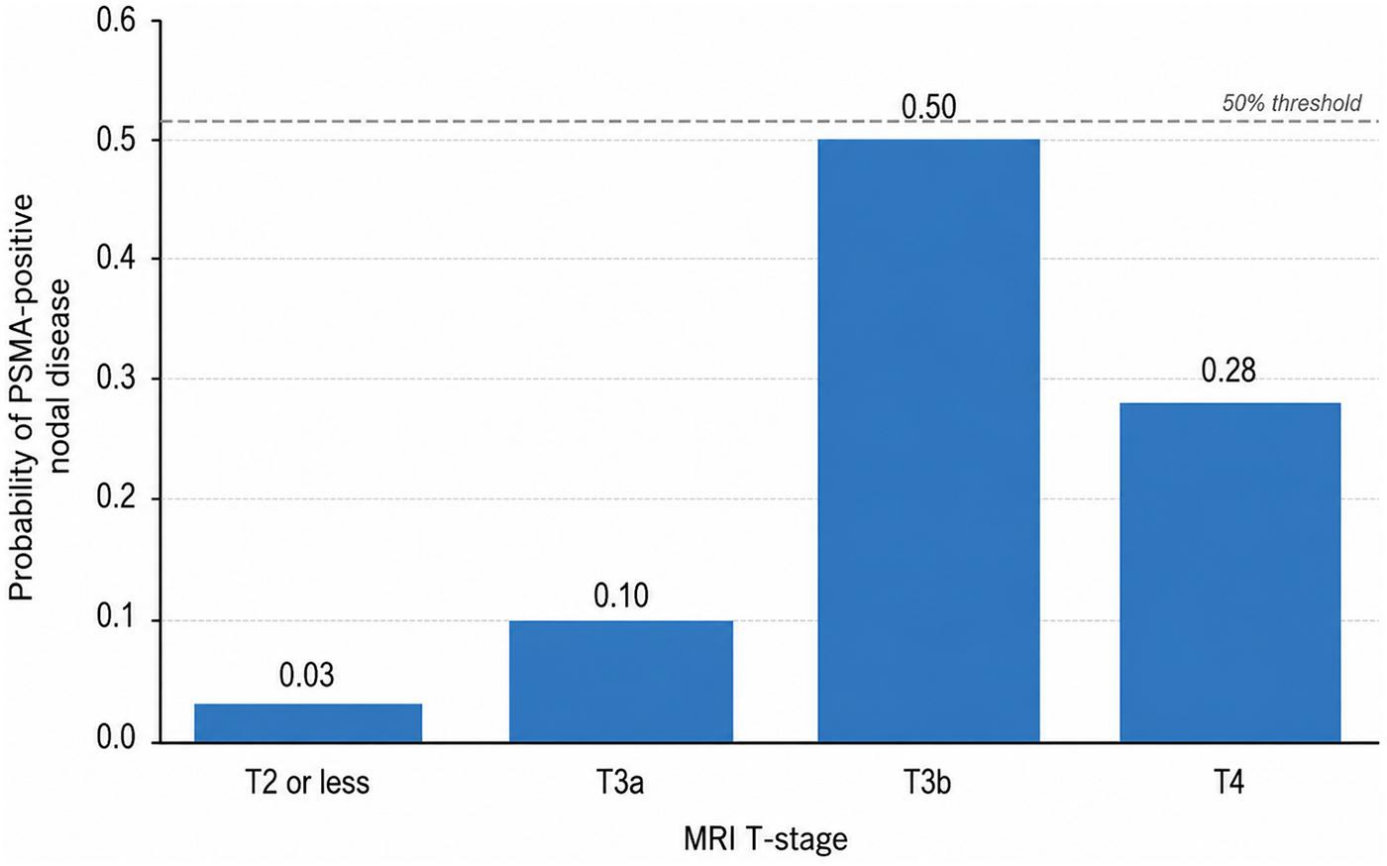
Predicted risk of PSMA-positive nodal disease by MRI-derived T stage. T3b disease was the only variable that independently conferred a greater than 50% predicted risk of PSMA-positive nodal disease.

The cohort includes older patients (age 75–80) with T2 or less disease, Gleason 7, and PSA typically below 50. To identify whether any single clinical variable alone could predict PSMA-positive nodal disease, we tested each factor individually while holding the others constant at low-risk levels; for example, PSA at 10, Gleason 7, and age 65. We found that MRI-derived T3b disease was the only variable that independently conferred a greater than 50% predicted risk of PSMA-positive nodal disease. Neither Gleason ≥8, high PSA, nor younger age alone were sufficient to cross this threshold unless combined with other risk factors (Figure 7).

## Discussion

In this retrospective cohort study of patients with high-risk primary prostate cancer, we demonstrate that MRI-derived T stage is associated with PSMA-positive nodal disease, with T3b disease emerging as the dominant independent predictor after adjustment for PSA, Gleason score and age. While T4 disease also showed an increased odds ratio of PSMA-positive nodal involvement, this finding is based on a small subgroup and should be interpreted cautiously, possibly attributable to selection bias in our cohort as patients referred for PSMA are those without metastases on conventional imaging. The wide confidence intervals associated with T3b disease indicate uncertainty regarding the precise magnitude of this effect, and T3a disease did not reach statistical significance in multivariable analysis (*p* = 0.086). These findings reinforce the critical role of MRI in pre-treatment risk stratification and suggest that local tumour extent, as assessed by MRI, may reflect a biological propensity for metastatic dissemination not captured by serum markers or histologic grade alone. These results align with prior evidence that advancing local tumour stage increases metastatic potential, and extend this concept specifically to PSMA-detected nodal disease (30–32).

The multivariable model incorporating MRI-derived T stage, PSA, Gleason score, and age demonstrated excellent performance, with an AUC of 0.863 (95% CI: 0.7947–0.916) and good calibration. Decision curve analysis confirmed that the model offers clinical net benefit across a range of risk thresholds, supporting its potential utility in guiding imaging decisions.

An important consideration is that PSMA PET/CT was used as the imaging reference standard for nodal disease rather than histopathologic confirmation. Accordingly, the present model predicts PSMA-positive nodal disease rather than true histologic nodal involvement. Nevertheless, PSMA PET/CT is increasingly incorporated into contemporary international guidelines as the preferred staging modality for high-risk prostate cancer because of its superior sensitivity and specificity compared with conventional imaging (33). Within this context, the present findings may help identify clinical/imaging phenotypes associated with particularly high or low likelihood of PSMA-positive nodal disease, potentially informing staging prioritisation and resource utilisation in settings where PSMA PET/CT availability remains limited.

We identified a small exploratory subgroup of patients in whom no PSMA-positive nodal disease was detected, defined by MRI-derived organ-confined disease (T2 or less), Gleason 7 histology, and PSA <20 ng/mL (*n* = 10). This cohort included both Gleason 3 + 4 (*n* = 7) and 4 + 3 (*n* = 3) disease. While this pattern is consistent with a very low likelihood of PSMA-positive nodal involvement on PSMA, the limited sample size necessitates cautious interpretation and these findings should be regarded as hypothesis-generating. Notably, all PSMA-positive nodal disease within the broader Gleason 7 cohort occurred in patients outside this low-risk definition, supporting the concept that not all guideline-defined “high-risk” patients share equivalent metastatic potential. This is particularly relevant given the heterogeneity of high-risk definitions across international guidelines, some of which include subsets of Gleason 7 disease within high-risk categories. The European Society of Nuclear Medicine for example includes primary pattern 4 + 3 disease in the definition of high risk prostate cancer (33), the American Urological Association cites Gleason score of ≥8 as high risk, but also includes ≥ cT2c as high risk (34). High risk in the Radiation Therapy Oncology Group classification includes (1) Gleason ≥8, or (2) Gleason = 7 plus either ≥ cT3 or node-positive (35).

Although PSMA PET/CT demonstrates excellent specificity for nodal metastatic disease, its sensitivity for small-volume or microscopic nodal disease remains imperfect (15, 36). Accordingly, the present model predicts PSMA-detectable nodal disease rather than all histopathologic nodal metastases. This distinction is particularly relevant when interpreting the observed exploratory low-risk subgroup, as occult microscopic nodal disease below the detection threshold of PSMA PET/CT cannot be excluded. Nevertheless, PSMA PET/CT currently represents the most sensitive clinically available staging modality for high-risk prostate cancer and is increasingly incorporated into contemporary guideline-based staging pathways.

Our data suggest that within this subgroup group, the absence of elevated PSA or extra-prostatic extension may identify a biologically favourable subset with minimal risk of PSMA-positive nodal disease on PSMA PET/CT and this subgroup may represent potential candidates for selective imaging de-escalation in resource-limited settings. Prospective validation in larger cohorts should be considered. In contrast, among all variables examined, MRI-derived T3b stage was the only factor that, when modelled in isolation, predicted a greater than 50% risk of PSMA-positive nodal disease. This finding underscores the dominant prognostic significance of local tumour extension and reinforces the role of MRI-derived T stage as a central determinant in risk stratification and potential triage for advanced molecular imaging in high-risk prostate cancer.

Prior studies have demonstrated that nomograms incorporating MRI-derived local staging parameters can improve prediction of pathologic lymph node metastases in prostate cancer (37). MRI-based nomograms integrating clinical stage, PSA, and Gleason score have shown improved discrimination compared with conventional clinical risk models alone. The present study builds upon this concept by specifically evaluating predictors of PSMA-positive nodal disease in a contemporary cohort undergoing PSMA PET/CT staging. Notably, MRI-derived T3b disease emerged as a particularly strong independent predictor of nodal positivity on PSMA PET/CT, suggesting that detailed MRI local staging may provide clinically relevant information for imaging-based nodal risk stratification and potential PSMA PET/CT triage. MRI-derived T stage emerged as an independent predictor of nodal disease on externally validated predictive models in other studies (38).

Exploratory multinomial regression revealed divergent patterns of PSMA-positive nodal dissemination. Gleason ≥8 and higher PSA were more closely associated with regional (N1) PSMA-positive nodal disease, while younger age emerged as a stronger predictor of distant (M1a) PSMA-positive nodal disease. While these findings may reflect distinct biological phenotypes, with aggressive regional progression vs. early systemic dissemination in younger patients, the underlying explanation however is uncertain and may reflect referral or selection bias within a retrospective PSMA PET/CT staging cohort rather than a true biological effect. While the mechanisms underlying these patterns remain unclear, they suggest that future risk models may benefit from differentiating between regional and distant nodal compartments.

We further examined the heterogeneity within Gleason 7 disease by stratifying patients into 3 + 4 and 4 + 3 subtypes. Although not statistically significant, PSMA-positive nodal disease were nearly three times more common in the 4 + 3 group (29.2%) than in the 3 + 4 group (10.3%), and the association persisted in multivariable analysis. Within this subgroup, MRI-derived T3 disease and elevated PSA demonstrated stronger associations with PSMA-positive nodal disease than Gleason pattern alone. These findings underscore the need for nuanced interpretation of intermediate-risk disease and support prior evidence that 4 + 3 pathology behave more aggressively than 3 + 4 counterparts (39–41).

Our study has limitations. First, its retrospective and single-centre design may limit generalisability. The cohort was appropriately restricted to high-risk prostate cancer, which aligns with the intended clinical application of PSMA PET/CT, the retrospective referral-based nature of imaging may introduce modest selection enrichment toward patients with more clinically concerning disease. Exclusion of 47 patients because of incomplete data may have introduced selection bias, which may affect generalisability of the observed associations and model performance. Although all imaging was performed and interpreted using standardised protocols, inter-reader variability in assigning MRI-derived T stage and PSMA positivity could influence findings. MRI-derived T-stage, particularly distinction between T3a and T3b disease, remains reader-dependent and may influence reproducibility across institutions and readers. False-positive PSMA uptake within benign ganglia and reactive lymph nodes is a recognised limitation of PSMA PET/CT interpretation. Biopsy techniques varied across the cohort and included both transrectal and transperineal approaches. As biopsy methodology was not fully standardised, sampling error and potential under- or over-estimation of Gleason score may have influenced risk stratification and model performance. Furthermore, although we applied bootstrap internal validation, the study remains limited by its retrospective single-centre design and the model has not yet undergone external validation in an independent cohort. The events-per-variable ratio was modest and bootstrap validation suggested some degree of overfitting. The reported performance metrics may overestimate performance and external validation with independent cohorts is required. Although we have outlined the imaging criteria used to assign MRI-derived T stage, the process remains partly subjective and dependent on reader experience and image quality. Some subgroups, particularly patients with T4 disease and the proposed exploratory low-risk combinations, were small, leading to wide confidence intervals and limiting the precision and generalisability of those estimates. While PSMA PET/CT is highly sensitive for nodal disease, microscopic disease below the detection threshold may still be missed.

## Conclusion

MRI-derived local staging, particularly T3b disease, was strongly associated with PSMA-positive nodal disease in this cohort of patients with high-risk primary prostate cancer. These findings suggest that MRI-derived T stage may provide clinically meaningful information for nodal risk stratification beyond traditional clinical variables alone and could potentially assist in guiding PSMA PET/CT staging pathways in selected patients. The absence of PSMA-positive nodal disease within a small exploratory low-risk subgroup warrants further investigation but should be considered exploratory. Although the proposed multivariable model demonstrated good discriminatory performance, external validation in larger multicentre cohorts is required before broader clinical implementation.

## Data Availability

The raw data supporting the conclusions of this article will be made available by the authors, without undue reservation.
